# Tumor suppressor BLU inhibits proliferation of nasopharyngeal carcinoma cells by regulation of cell cycle, c-Jun N-terminal kinase and the cyclin D1 promoter

**DOI:** 10.1186/1471-2407-12-267

**Published:** 2012-06-22

**Authors:** Xiangning Zhang, Hui Liu, Binbin Li, Peichun Huang, Jianyong Shao, Zhiwei He

**Affiliations:** 1 Department of Pathophysiology, Guangdong Medical College, 1 Xincheng Road, Song-Shan Lake (SSL) Science Technology and Industrial Park Dongguan, Guangdong, 523808, China; 2 Key Laboratory for Medical Molecular Diagnostics of Guangdong Province Sino-American Cancer Research Institute, Guangdong Medical College, 1 Xincheng Road, Song-Shan Lake (SSL) Science, Technology and Industrial Park, Dongguan, Guangdong, 523808, China; 3 State Key Laboratory of Oncology in South China, Department of Molecular Diagnostics, Sun Yat-sen University Cancer Center (SYSUCC), 651 Dong Feng Road, Guangzhou, Guangdong, 510060, China

**Keywords:** Nasopharyngeal carcinom, BLU/ZMYND10, Cell cycle, JNK, Cyclin D1

## Abstract

**Background:**

Tumor suppressor genes function to regulate and block tumor cell proliferation. To explore the mechanisms underlying the tumor suppression of *BLU*/*ZMYND10* gene on a frequently lost human chromosomal region, an adenoviral vector with *BLU* cDNA insert was constructed.

**Methods:**

*BLU* was re-expressed in nasopharyngeal carcinoma cells by transfection or viral infection. Clonogenic growth was assayed; cell cycle was analyzed by flow cytometry-based DNA content detection; c-Jun N-terminal kinase (JNK) and cyclin D1 promoter activities were measured by reporter gene assay, and phosphorylation was measured by immunoblotting. The data for each pair of groups were compared with Student *t* tests.

**Results:**

*BLU* inhibits clonogenic growth of nasopharyngeal carcinoma cells, arrests cell cycle at G1 phase, downregulates JNK and cyclin D1 promoter activities, and inhibits phosphorylation of c-Jun.

**Conclusions:**

*BLU* inhibits growth of nasopharyngeal carcinoma cells by regulation of the JNK-cyclin D1 axis to exert tumor suppression.

## Background

Tumor suppressor genes (TSGs) are implicated in the genesis of cancer following loss of function, and such losses normally contribute to deficiencies in cell cycle modulation and apoptosis induction. These losses of cell function are usually either due to homozygous deletion or hypermethylation on promoter regions [1], which would normally function to inhibit cell proliferation and thereby suppress tumor growth. The short arm of human chromosome 3 (3p) harbors a 670 kb region, which is frequently deleted in various cancers. A minimal candidate region of 120 kb has been identified, and the affected genes include Ras-associated factor 1 (*RASSF1*), and β-catenin in lung cancer (*BLU*) [2,3].

Nasopharyngeal carcinoma (NPC) is a cancer of head and neck squamous cells, and is endemic in southern China and certain regions of Southeast Asia. Its occurrence involves the interaction of host genetic materials with environmental factors, notably infection by Epstein-Barr virus (EBV). EBV plays a crucial role in the clonal expansion of pre-malignant cells [4], and regionally prevalent viral strains may be responsible for carcinogenesis [5]. Genetic or epigenetic changes of *BLU* are frequent in NPC [6-8].

RASSF1 isoform A (*RASSF1A*), located on the 3p21 region, is one of the TSGs clustered on the 120 kb fragment affected during the genesis of human tumors, as described above. It codes for a zinc finger protein and transcriptionally regulates a panel of genes to modulate apoptosis and cell cycle [9]. Ectopic expression of *RASSF1A* inhibits cyclin D1 and arrests the cell cycle in the G1 phase [10]. C-Jun N terminal kinase (JNK) is a member of the mitogen activated kinase (MAPK) family. It is activated by extracellular stress and other stimuli, and in turn triggers a kinase cascade and contributes to the formation of dimeric transcription factor AP1 on phosphorylating c-Jun. In line with the expression of cyclin D1 induced by c-Jun [11], the downregulation of cyclin D1 is attributed to JNK inhibition by RASSF1A [12].

Studies have shown that *BLU* is lost in various human tumors. When transfected into HONE-1 cells derived from NPC, *BLU* inhibits the tumorigenesis of nude mice xenografts [13]. This result suggests that expression of *BLU* downregulates cell proliferation either through apoptosis or by cell cycle regulation. The candidate TSGs from the 3p region were transferred to lung cancer lines to evaluate their tumor suppression. Some of these genes have the potential to induce apoptosis upon expression. Apoptosis, however, was not observed in *BLU*-expressing cells [14]. *BLU* encodes a protein of 440 amino acid residues, which has a zinc finger MYND (myeloid Nervy deformed epidermal auto-regulatory factor-1 (DEAF-1)) domain, ZMYND [3]. ZMYND domain-containing proteins define a protein family whose members associate with molecules such as co-repressors to regulate transcription and may modulate the process of malignant transformation. BLU is alternatively termed ZMYND10, and is structurally similar to ZMYND2 [2,3]. ZYMND2 forms a fusion protein with acute myeloid leukemia-1 (AML-1) due to a t(8;21) chromosomal translocation, which is the most frequent chromosomal aberration seen in acute myeloid leukemia. AML-1-dependent transactivation is inhibited by fusion with ZMYND2 (also known as ETO) [15].

The ZMYND is the only functional domain present within the BLU protein molecule, and it is a potential binding site for co-repressors of transcription, such as nuclear co-repressor (NcoR), mSin 3A, and the histone deacetylases [16]. The ability of *BLU* to regulate transcription is speculated to influence the level of proliferation regulators, and engagement of signaling pathways involving JNK and cyclin D1 may mechanistically elucidate the tumor suppressive role played by *BLU*.

## Methods

### Cells and plasmids

CNE-1 and CNE-2 lines derived from well differentiated and undifferentiated NPCs from Chinese patients, respectively, were either obtained from the nitrogen stock in our department or purchased from the Cell Bank, Institute of Life Science, Chinese Academy of Science, Shanghai, China. The esophageal cancer line EC109 was obtained from the laboratory of Professor Qian Tao, Chinese University of Hong Kong. The full-length cDNA of *BLU*/*ZMYND10* (GenBank accession no. NM_015896) (Additional file 1)was chemically synthesized and inserted into the SalI-SacI sites of the pCD316-EGFP plasmid (Viral Gene Transfer Company, Beijing, China), and the inserted fragment was confirmed by sequencing. *BLU* and *TP53* inserted in the pcDNA3.1 vector were provided by Professors Qian Tao and Bert Vogelstein, Johns Hopkins University, Baltimore, MD, USA, respectively. The reporter plasmid pRTU14 with an AP-1 region located upstream of the coding gene of luciferase was a gift from Dr Arnd Kieser, Helmholtz Zentrum München, Munich, Germany [17], and a cyclin D1 promoter reporter plasmid-1745 CD1 LUC containing the full-length cyclin D1 gene promoter was a kind gift from Dr Richard Pestell, Thomas Jefferson University, Philadelphia, PA, USA [11]. The control reporter plasmid, pRL-TK expressing *Renilla* luciferase, was purchased from Promega (Beijing, China).

### Antibodies and reagents

Goat anti-human BLU/ZMYND10 protein antibody was purchased from Abcam (Cambridge, UK). Rabbit anti-c-Jun and anti-phospho-c-Jun (Ser73) antibodies, and anti-cyclin D1 antibody were purchased from Cell Signaling Technology (Danvers, MA, USA). Mouse anti-human actin monoclonal antibody (mAB) and clone C4 was purchased from Millipore (Beijing, China). Ultra Red Odyssey-labeled rabbit anti-mouse and rat anti-rabbit immunoglobulins were purchased from Invitrogen (Guangzhou, China). Transfection reagent FuGene HD was purchased from Roche (Shanghai, China). Luciferin substrate and stop reagent were purchased from Promega (China).

### Production of Ad-*BLU*-enhanced green fluorescence protein (EGFP) virions and infection of NPC-derived cells

Ad-*BLU*-EGFP virions were prepared by transfection of pCD316-*BLU* to packaging 293 cells. CNE2 cells were incubated with different doses of the viral stock, and grown for 48 h on sterile cover slips placed on the bottom of six-well culture plates.

### Confocal microscopy

At the time of harvest, cover slips seeded with transfected cells were washed with phosphate-buffered saline (PBS), fixed with 1:1 acetone-methanol, and stained with propidium iodide (PI; Invitrogen), and then mounted on slides with medium containing an antifade reagent. Images were captured at × 400 magnification using Laser Sharp Software (Macrologic Solutions, Albuquerque, NM).

### Transfection

CNE-2 cells were seeded in 6- or 12-well culture plates, and incubated at 37°C with 5% CO_2_. Cells were then transfected with 0.5 μg (12-well plates) or 1 μg (six-well plates) DNA by mixing with FuGene HD, as indicated in the manual provided by the manufacturer. Cells were then incubated for 24 h.

### Colony formation inhibition assays

Assays were conducted as previously described [18]. A total of 1 × 10^5^ CNE2 or EC109 cells were seeded in 12-well plates, and were transfected with pcDNA3.1-*BLU*, pcDNA3.1-*TP53*, empty vector pcDNA3.1 or were left untransfected. After 48 h, 1 × 10^4^ cells were seeded in triplicate in 6-well culture plates, and cultured with complete medium containing 500 μg/ml G418 (Gibco Biotechnology) for 2 weeks. Cells were then fixed with ice-cold acetone-methanol (1:1), stained with gentian violet and washed with sterile PBS. Colonies containing more than 50 cells were counted, and comparisons were made between *BLU*-, *TP53*- and mock-transfected and untransfected cells. The experiment was repeated at least three times, and the data were evaluated statistically.

### Luciferase assays

CNE-2 cells were co-transfected with expression vectors (*BLU* or empty vector), JNK or cyclin D1 promoter reporters, and the internal control plasmid TK-RL for 24 h, and then harvested by lysis with 1× Passive Lysis Buffer (Promega, China). Lysate was added to each well of a non-transparent 96-well plate and mixed with luciferin. The luciferase activity was measured with a BioTek ELISA reader, followed by measurement of the firefly luciferase activity. Luciferase activity was then quenched with GLO Stop reagent, and new luciferin was added. The data were analyzed with Gen 5 Wizard software (BioTek, Beijing, China). The ratios of the two readings were calculated and interpreted as the calibrated reporter activity of the transcription gene. The data were presented as mean ± standard deviation (SD), and were derived from at least three independent experiments.

### Flow cytometry

Monolayers of up to 2 × 10^5^ cells transfected with pCD316-*BLU* M PI diluted with staining buffer (100 mMTris, pH7.4, 150 mM NaCl, 1 mMCaC1_2_ or empty vector were grown on 6-well culture plates for 24 h or 48 h. Following harvest, they were fixed with absolute ethanol, stained with 3 μ2 M PI diluted with staining buffer (100 mM Tris, pH7.4, 150 mM NaCl, 1 mM CaCl_2_, PI diluted with staining buffer (100 mM Tris, pH 7.4, 150 mM NaCl, 1 mM CaCl2 , 0.5 mM MgCl2, 0.1% Nonidet P-40) and detected using a FACS Canto (Becton Dickinson, San Jose, CA, USA). The DNA content of the cells was analyzed using Win Cycle32 software (Phoenix Flow Systems Inc., San Diego, CA, USA).

### Western blotting

Transfected cells were pelleted and dissolved in 2× loading buffer (130 mM Tris, pH 6.8, 4% sodium dodecyl sulfate (SDS), 20% glycerol, 10% mercaptoethanol). Total protein was separated by SDS-PAGE and electro-blotted to nitrocellulose membranes, then probed with appropriate primary antibodies diluted in blocking buffer at 1: 500 (Goat anti-human BLU; Abcam) or 1: 1,000[Rabbit anti-c-Jun and anti-phospho-c-Jun (Ser73) antibodies, and anti-cyclin D1 antibody, CST)] with 5% nonfat milk in PBS overnight at 4°C or 1 h at room temperature. After washing with 0.1% Tween-20 in PBS, membranes were incubated with secondary antibodies at 1:10,000 dilution. Blots were developed in an Odyssey Infrared Imager (LI-COR Bioscience, Lincoln, NE, USA).

### Statistical analysis

All quantitative data were obtained from three independent tests, and presented as mean ± SD. Student *t*-tests were used to compare the mean values of each group, and in all cases, *P* <0.05 was considered statistically significant.

## Results and discussion

**Figure 1:**
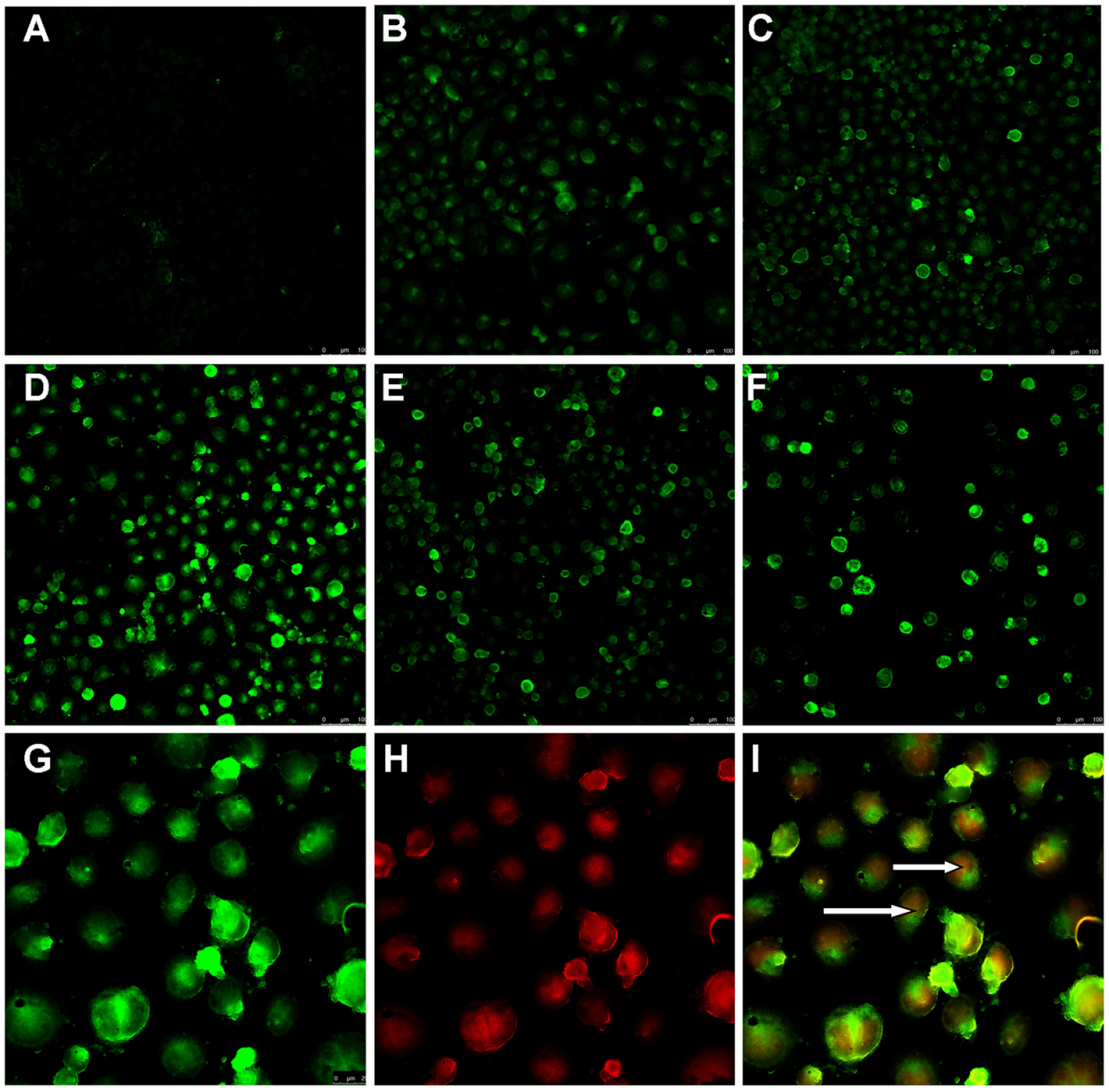
***BLU*expression in CNE-2 cells following adenovirus-mediated gene transfer.** (**A**–**F**) CNE 2 cells plated in six-well culture plates were challenged with 0, 10, 50, 100, 200, or 400 PFU viral particles. (**G**-**I**) Cells were grown on cover slips, fixed and viewed with a confocal microscope at a resolution of × 100. The cells were identically manipulated, stained with 3 M PI, and viewed with confocal microscope at a resolution of × 400 for EGFP. The overlapping signals were analyzed on merged figures. The arrows indicate cytoplasmic localization of BLU protein.

*BLU* was efficiently transferred by adenoviral vector to CNE2 cells. To study the tumor suppressive effects of *BLU*, cDNA was chemically synthesized and inserted into pCD316 adenoviral expression vector. After transfection into a packaging cell line 293, a total amount of 1 × 10^12^ viral particles/ml were obtained. Two NPC derived lines, CNE-1 and CNE-2 were chosen to study the effect of ectopic expression of BLU on the growth of NPC because the BLU expression is lost in these cells due to the presence of promoter hypermethyaltion [8]. BLU has been shown to be downregulated in other lines, but those lines still exhibit basal expression, for example, the EBV positive C666-1 [8]. Supernatant containing virions, termed Ad *BLU*, with 10, 50, 100, 200, or 400 plague-forming units (PFU) per cell, as previously described [19], were co-incubated with CNE-2 cells. Cells were transfected with EGFP fused with the target gene, and there was no cytotoxicity at a dose of 100 PFU per cell. Thus, this dose was considered optimal for *BLU* transfer (Figure 1A–F).

In contrast, the results obtained for determination of the optimal infection dose of Ad *BLU* in CNE-1 cells differed: (Additional file 2) these data are to be further confirmed using other approaches before *in vivo* studies are performed. Counterstaining with PI revealed a mixed pattern of subcellular distribution, i.e. cytoplasmic and nuclear localization of overexpressed *BLU*. Our results were in line with the Gene Ontology data (http://www.uniprot.org/uniprot/O75800), suggesting predominant cytoplasm distribution (Figure 1F–I).

### Inhibition of clonogenic growth and cell cycle entry by expression of *BLU* in CNE-2 cells

**Figure 2:**
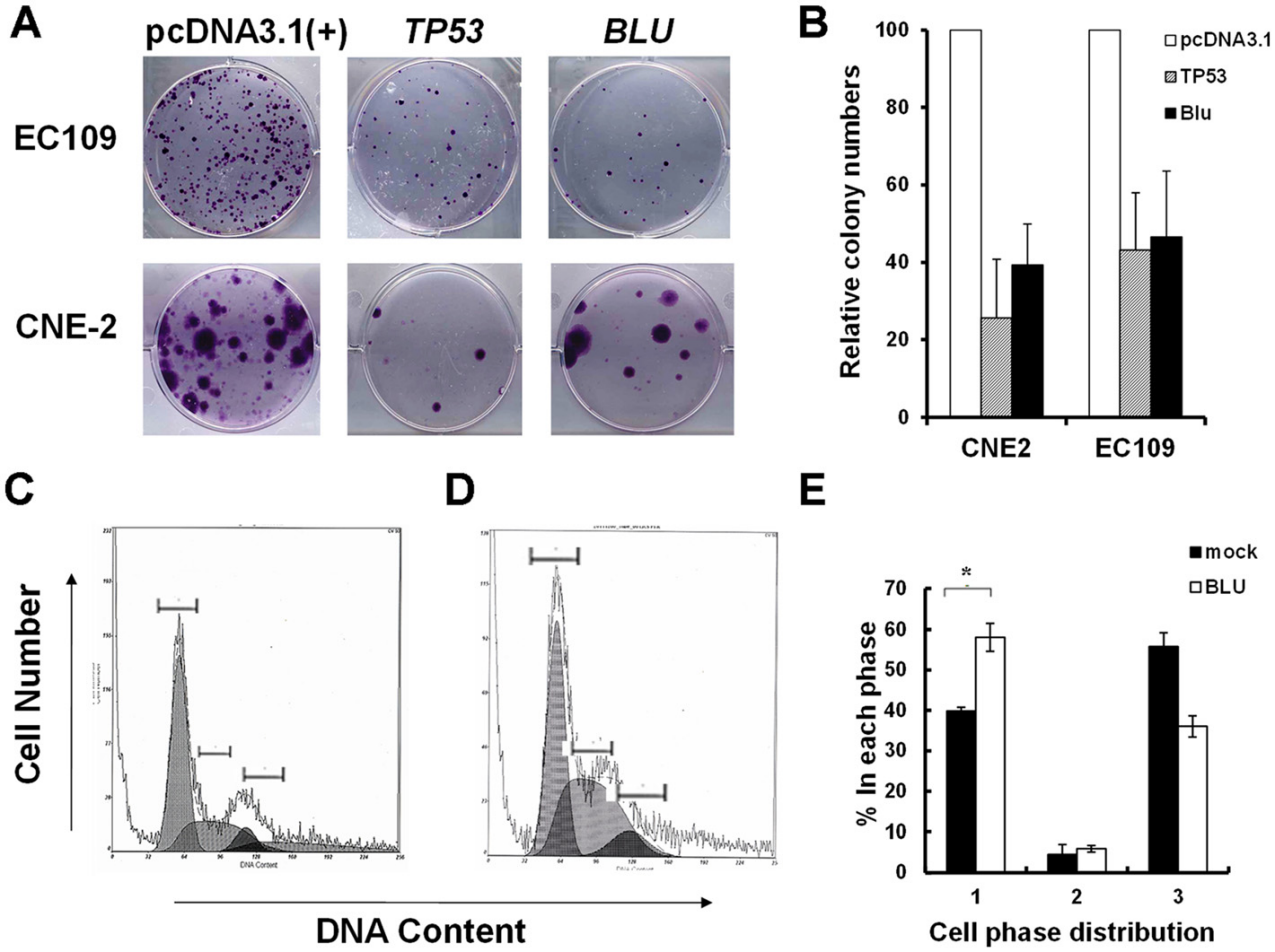
***BLU*expression exerts growth inhibitory activity and cell cycle arrest.** (**A**) NPC-derived CNE-2 line and esophageal carcinoma-derived EC109 line were transfected with empty pcDNA3.1 vector, tumor suppressor *TP53* or *BLU*. Cells were cultured in complete medium supplemented with 500 μg/ml G418 for up to 2 weeks, harvested and stained with gentian violet. (**B**) Summary of the results in (A). The colonies obtained for each experimental condition were counted and the numbers were presented as mean ± standard deviation. (**C**) and (**D**) CNE-2 cells were transfected with vector or pCD316-*BLU* and stained with PI diluted in assay buffer. The DNA contents were measured with FACS and analyzed with Win Cycle32 software. (**E**) Results of FACS analysis. The numbers 1, 2, and 3 depict the populations of cells in G1, G2/M and S phases. BLU expression arrested the cells in G1 phase, as manifested by the increase in this population. The data is presented as mean ± SD derived from at least three independent experiments.

Both *BLU*-expressing plasmid and recombinant *BLU* adenovirus Ad *BLU* were used in the present study. The potential inhibition of growth by *BLU* was assayed with pcDNA3.1 vector, and the effect was compared with that of the tumor suppressor *TP53* expressed from the same eukaryotic vector. The effect was tested in two malignant lines of epithelial origin. CNE-2 cells are an NPC line that is negative for *BLU* expression [8], and EC109 cells were derived from an esophageal cancer patient in which the expression of *yh* is absent. Ectopic expression of *BLU* significantly suppressed the number of colonies by more than 50% of the mock transfected cells comparable with the extent seen in *TP53*-transfected cells (Figure 2A), implying suppression of tumorigenicity by *BLU*.

Tetracycline-regulated *BLU* suppresses tumor formation, while downregulation of *BLU* can promote tumor growth in nude mice with human NPC xenografts [13]. However, forced expression of *BLU* in lung cancer cells fails to induce apoptosis [14]. Upon staining with DNA dye, PI-fragmented nuclei were not visible by confocal microscopy (Figure 1A–I). The suppression of cell proliferation could be promoted by blocking cell cycle entry. To identify the nature of the cell cycle arrest, CNE2 cells were transfected with pCD316-*BLU* and empty vector, and examined for DNA content by FACS. Analysis of PI incorporation shows that in comparison to the mock transfected cells, the majority of CNE-2 cells expressing *BLU* were in the G1 phase of the cell cycle (Figure 2D–F).

### *BLU* acts on JNK signaling to regulate cyclin D1 expression

**Figure 3:**
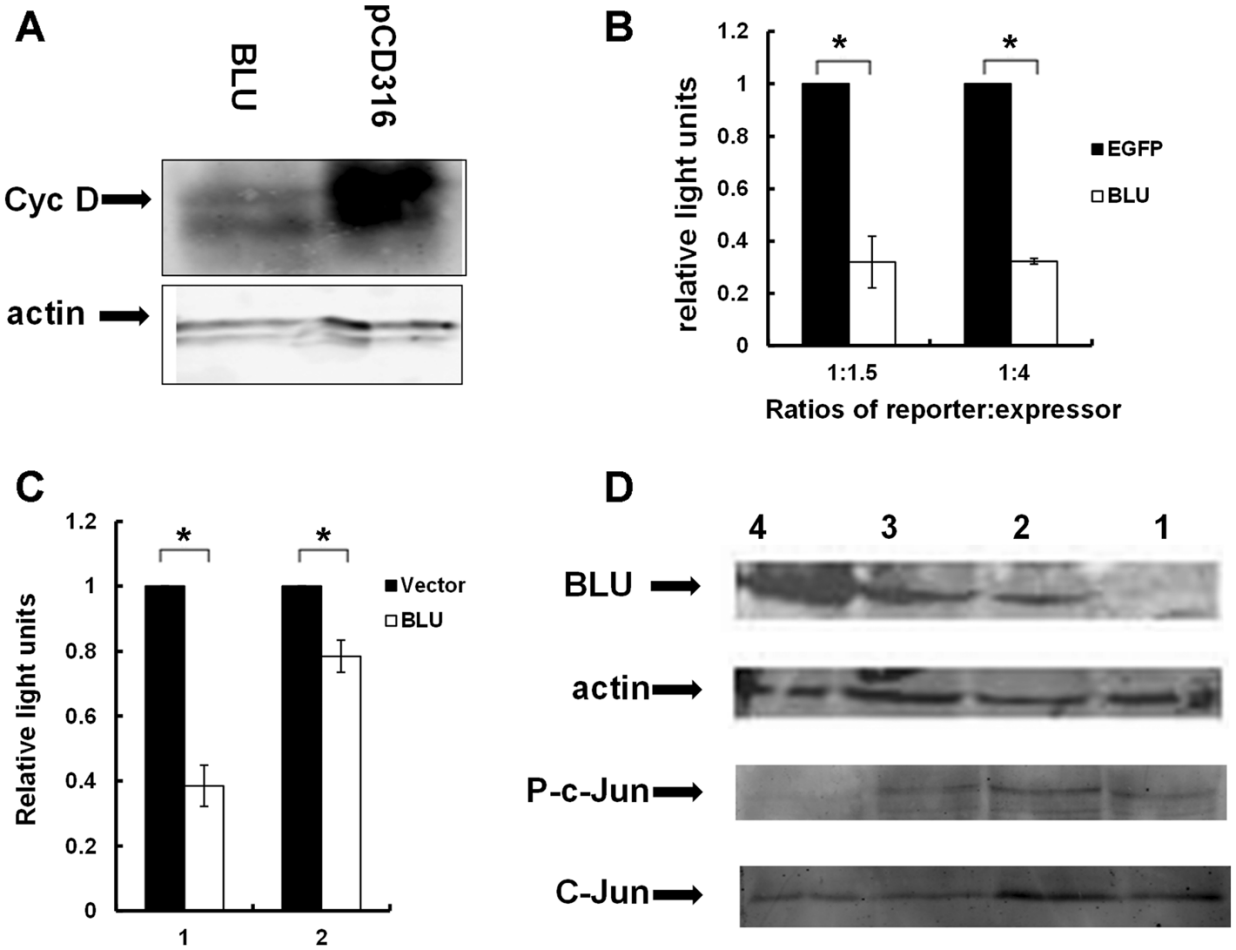
***BLU*expression downregulates cyclin D1 promoter and JNK activity, and inhibits phosphorylation of c-Jun**. (**A**) CNE-2 cells were transfected with pCD316 vector or pCD316-*BLU*, and cell lysates were immunoblotted and probed with goat anti-human BLU polyclonal antibody. The membranes were stripped and re-probed with anti-actin mAb clone C4. (**B**) Expression of BLU inhibits cyclin D1 promoter activity. pCD316 and pCD316p-*BLU* plasmids were co-transfected with cyclin D1 promoter reporter at concentration ratios of 1:1.5 and 1:4, respectively. The luciferase activity was measured for the two conditions, and the reporter activity was presented as the ratio of the two. The data are presented as the mean ± SD, and are derived from at least three independent tests. *Indicates *t* < 0.05 when compared with the measured values from the two groups. (**C**) pCD316-*BLU* and empty vector were co-transfected with JNK reporter at concentration ratios of 1:1.5 and 1:4, and the reporter activity was calculated. The data re presented as mean ± SD, and were derived from at least three independent tests. *Indicates *P* < 0.05 when comparing the calculated values for the relative light units between the two groups. (**D**) CNE-2 cells were infected with 0, 10, 50 and 100 PFU Ad *BLU* (lanes 1, 2, 3, 4), and ectopic expression was demonstrated by immunoblotting and probing with anti BLU goat polyclonal, anti actin mAb, anti phospho-c-Jun (P-c-Jun) and anti-c-Jun rabbit polyclonal antibodies. c-Jun phosphorylation was inhibited by infection with 100 PFU Ad *BLU*.

Cyclin D1 protein is involved in the process of permitting cells to enter S phase. Consistent with its role in regulating G1/S phase progression, *BLU* expression dramatically reduced cyclin D1 level (Figure 3A). This is in contrast to previous findings in RASSF1A-ectopically expressing cells, in that regulation of the cyclin D1 promoter does not appear to be inhibited upon *RASSF1A* expression [11]. However, expression of *BLU* in CNE-2 cells significantly inhibited the activity of the cyclin D1 promoter, as manifested by the different levels of the luciferase gene co-expressed with pCD316-*BLU* (Figure 3B). Transcription of cyclin D1 has been suggested to be regulated by the c-Jun transcription factor, because of the presence of a c-Jun activation site on its promoter [11]. Modulation of JNK activity by *BLU* was tested by co-transfection of the AP1 reporter, in which luciferase expression is driven by activated JNK. We found that *BLU* dramatically blocks the reporter (Figure 3A) and leads to the inhibition of c-Jun phosphorylation at a dose of 100 PFU per cell (Figure 3C and D).

## Conclusions

In recent years, human adenovirus (Ad) has been widely used as a vector for gene transfer to mammalian cells, owing to its ability to effectively infect a wide variety of cells [20]. Replication deficient viruses proliferate for weeks in hosts with cancer or other diseases, enabling the restored expression of a defected gene to achieve therapeutic goals. In the present study, a putative tumor suppressor *BLU* was efficiently transferred to an NPC-derived line CNE-2. CNE-2, together with cell lines of identical histological origin, i.e. undifferentiated tumors arising in the nasopharynx from the endemic region, exhibit downregulated *BLU* expression as a result of promoter methylation [8]. A dose-dependent infection efficiency was observed.

The genesis of cancer is a multi-step event, and aberrations involving genes on the 3p21 region, including homozygous deletions and promoter hypermethylation affecting cluster of TSGs, are early molecular changes in various human tumors [2]. Re-expression of the lost TSGs complementary to other interventions, for example, enhancement of host immune surveillance, contributes to the improvement of currently available anti-cancer modalities. Our result prompts further efforts to demonstrate the potential of transferred *BLU* gene to inhibit *in vivo* tumor formation. The system could be improved by replacing the ubiquitous promoter derived from murine cytomegalovirus with a regulatory element that has high activity in the malignancies to control the expression of biotherapeutic target genes. Such elements include α-fetoprotein enhancer-promoter for hepatocellular carcinoma cells [21], and origin of plasmid replication (OriP) element [22,23].

The current data, in part, mechanistically explain *BLU*-mediated tumor suppression. It was demonstrated in the present study that *BLU* inhibited clonogenic growth of NPC and esophageal cancer cells in which endogenous expression of *BLU* is absent. The inhibition of colony formation may be due to the ability of *BLU* to induce apoptosis or prevent cell cycle progression. TSGs from the 3p region that were adenovirally transferred to lung cancer cells were tested for their potential to induce apoptosis. While some genes indeed showed potential to induce apoptosis, *BLU* had no effect on the initiation of apoptosis [14]. Similar with this previously published data [14], the NPC cells did not undergo apoptosis even when exposed to high doses of Ad *BLU*, as evidenced by no visible characteristic changes.

Therefore, growth inhibition by *BLU* may result from the blocking of cell cycle entry. The entry to S phase from G1 is controlled by cyclin D1 and other cell cycle regulators. We have shown that cyclin D1 levels are significantly reduced in *BLU*-expressing cells. Activation of the cyclin D1 promoter in response to extracellular signals is dependent on the binding of its AP1-like sequence following different stimuli. It has been reported that the cyclin D1 gene promoter is induced by c-Jun [11], but it remains to be tested whether *BLU* regulates the upstream kinase of c-Jun, JNK. Reporter assays revealed that similar to *RASSF1A*, *BLU* inhibits JNK activity, leading to its lower phosphorylation catalytic activity. An upstream kinase which regulates the activity of JNK-AP1 axis, apoptosis signal regulated kinase 1 (ASK1) has been shown to be transcriptionally regulated by RASSF1A [9]. It has been suggested that that tumor suppressors proteins with zinc finger domains may regulate cell proliferation via transcription regulation. The reduced levels of cyclin D1 may be due to transcriptional downregulation, as BLU also inhibits the activity of its promoter, in contrast to the inhibition of its accumulation by RASSF1A [10]. JNKs have been shown to serve as mediators of apoptosis in response to cellular stress to sustain cell proliferation [17], and have been implicated in survival in response to extracellular stimuli such as cytokines. They are serine/threonine protein kinases that can be activated by a variety of stimuli including environmental stress (UV and ionizing radiation, heat shock, osmotic or redox shock), inflammatory cytokines, and growth factors. Different species of zinc finger proteins function to regulate gene transcription, similar with ZNF418 [24], a member of the zinc finger transcription factor family. BLU may negatively regulate gene transcription mediated by the MAPK signaling pathways through transcriptionally regulating a molecule(s) yet to be identified. It has been reported that the *BLU* promoter is stress-responsive [8], and this implies an interaction between BLU and stress-induced JNK. In addition, the effects of BLU on the upstream signaling molecule of the JNK pathway remain to be explored.

It is therefore speculated that BLU inhibits JNK activity by transcriptional repression mediated through its ZMYND domain, which is closely associated with co-repressors such as nuclear co-repressor (NcoR), or histone deacetylase, to regulate gene transcription [16]. The present study revealed that JNK and cyclin D1 are targets of tumor suppression exerted by BLU.

In conclusion, a putative tumor suppressor, *BLU*, plays a vital role in growth inhibition in NPC malignancies via JNK and cyclin D1 promoter inhibition.

## Abbreviations

NPC: Nasopharyngeal carcinoma; EBV: Epstein-Barr virus; TSG: Tumor suppressor gene; ZMYND: Zinc finger myeloid nervy deformed epidermal auto regulatory factor-1 containing domain; JNK: c-Jun N-terminal kinase.

## Competing interests

The authors declare that they have no competing interests.

## Authors’ contributions

XZ designed and performed the experiments, analyzed the data and drafted the manuscript. HL and LB contributed experimental reagents, performed experiments, and analyzed the data. JS and PC participated in the experimental design and analyzed the data; ZH designed the experiments, analyzed the data and drafted the manuscript.

## Pre-publication history

The pre-publication history for this paper can be accessed here:

http://www.biomedcentral.com/1471-2407/12/267/prepub

## Supplementary Material

Additional file 1 Tumor suppressor BLU inhibits proliferation of nasopharyngeal Carcinoma cells by regulation of cell cycle, JNK and cyclin D1 promoter. Click here for file

Additional file 2 Transferred Tumor suppressor BLU inhibits proliferation of nasopharyngeal Carcinoma cells by regulation of cell cycle, JNK and cyclinD1 promoters. Click here for file
