# Supplementary material for: Tumor suppressor BLU inhibits proliferation of nasopharyngeal carcinoma cells by regulation of cell cycle, c-Jun N-terminal kinase and the cyclin D1 promoter
Source: BMC Cancer. 2012 Jun 22;12:267. doi: 10.1186/1471-2407-12-267 (PMC3585814; doi:10.1186/1471-2407-12-267)
Supplement: Additional file 1 — Tumor suppressor BLU inhibits proliferation of nasopharyngeal Carcinoma cells by regulation of cell cycle, JNK and cyclin D1 promoter. [file 1471-2407-12-267-S1.docx]

Title: Transferred Tumor suppressor BLU inhibits proliferation of nasopharyngeal

Carcinoma cells by regulation of cell cycle, JNK and cyclin D1 promoters

Version: 1 Date: 12April2012

Reviewer: LifuHu

Reviewer's report:

The authors try to explore the mechanism of tumor suppressor gene BLU in NPC and

found this gene could arrest cells at G1 phase, down-regulates activity of JNK and D1

promoter and inhibits phosphorylation of c-Jun molecule.

There are some comments for this manuscript.

1. Down-regulationof Blu gene,in most case, is due to promoter methylation and

LOH in NPC. Homozygous deletion is arare event in tumors.

How is the different between CNE1 and CNE2 after transfection with Blu gene,

subcellular distribution or localization? Since most of undifferentiated NPC are

EBVpositive, where EBNA1, LMP1/LMP2 and EBERs are expressed, EBV positive

NPC cell line C6661 would be a good target cell to solve the differences resulting

from CNE1 and CNE2.

According to different line of experiments using different vector systems, we

have observed a time cource dependent cytoplasm to nuclei translocation of BLU.

But as stated in the present MS, the distribution of BLU coding product was

predominantly cytoplasmic in line with the online data. Its relation to the

protein function is unclear. The suggestion of using C666-1 line is very

intriguing in light of the tight association of EBV infection with occurrence of

undifferantiated NPC. C666-1 line, the only known EBV positive NPC and a

good system closely resembling the in vivo tumor, however, was not considered

when the experiments were designed because it had basal expression of BLU

according to the BLU expression profile of NPC cell panel reported by Qian Tao

and collaborators (Qiu et al., Oncogene 2004, 23:4793-4806. ref 8 in the present

MS.). We added the presription to the text. For further work we plan to deal

with the specific issue, by restoring EBV carrying status of our NPC cells.

2. Figure legend is not well described.For example,in Fig1,F to I. In Fig 2, There is

no marker by A , B C, D and E. It needs to point out Fig.2C for vector and Fig 2G

for Blup CD316.

Thanks the referee for pointing out such important points. The errors could be

generated by file transfer. As the labels were clear in our draft. The

Generated by Foxit PDF Creator © Foxit Software

http://www.foxitsoftware.com For evaluation only.corrections were made accordingly: Fig 2 A: Result of clonogenic growth

inhibtion; Fig. 2B: the summary of the data in (A). Fig. 2C, the cell cycle profile

of mock transfectant of CNE-2 with vector pCD316; and Fig. 2D, that of BLU

transfectant CNE-2.

3. In result part of“Blu acts JNK signaling to regulate cyclin D1 expression ”

D, CNE-2 cells were infected with 0,10,50 and 100 PFU AdBLU. How they were

Organized in Fig3 D by marker, from left (100PFU) in lane1 to right (0) in lane 4?

The trend of result is clear, but the quality of Western blot is poor. In the same result

part, ” It has been observed that BLU dramatically blocks the reporter (Fi 3A”. How

can we see this effect in Fig 3A, and leads to inhibition of phosphorylation of c-Jun at

dese of 100PFU per cell (Fig3C and 3D)?

To clarify here, the labels were listed, from left to right, 1, 2, 3, 4, and the

numbers corresponded with doses 0, 10, 50 and 100 PFU. The polyclonal

antibody(s) always generated such problems of high background. The present

figure displayed the one with the clearest dose dependency, as pointed out by the

referee. We are sorry for the quality problem of the blot as criticized by the

referee, but we have repeated the experiment for a number of times.

As stated in the subtitle in result part, the Figure 3 deal with the downregulation

of cyclin D1 by BLU. BLU expression reduced the level of cyclin D1 (Fig. 3A);

and inhibited the cyclin D1 promoter (Fig. 3B); A signaling axis has been

postulated to exist between JNK and cyclin D1, Fig. 3C and D showed that BLU

inhibited both JNK reporter and catalytic activities.

4. In discussion part, the first sentence,how the aberrations involving the genes On

3P21 region, including homozygous deletions and promoter hypermethylation

could affect the cluster of T SGs? In general, this manuscript provided some

evidences for mechanism of TSG BLU gene to inhibit cell proliferation on NPC

growth. It could be published after making correction, particularly polish in

English.

Generally we thank the referee for his encouragement, to will try our best to

improve the MS. Although the LOH has been reported to involve multiple gene

on the 3p21 region, to our knowledge, methylation may not affect all the TSGs on

the same region. We would remove the phrase “ the cluster of”…

Generated by Foxit PDF Creator © Foxit Software

http://www.foxitsoftware.com For evaluation only.
