# Supplementary material for: Tumor suppressor BLU inhibits proliferation of nasopharyngeal carcinoma cells by regulation of cell cycle, c-Jun N-terminal kinase and the cyclin D1 promoter
Source: BMC Cancer. 2012 Jun 22;12:267. doi: 10.1186/1471-2407-12-267 (PMC3585814; doi:10.1186/1471-2407-12-267)
Supplement: Additional file 2 — Transferred Tumor suppressor BLU inhibits proliferation of nasopharyngeal Carcinoma cells by regulation of cell cycle, JNK and cyclinD1 promoters. [file 1471-2407-12-267-S2.docx]

Reviewer's report

Title: Transferred Tumor suppressor BLU inhibits proliferation of nasopharyngeal Carcinoma cells

by regulation of cell cycle, JNK and cyclinD1 promoters

Version: 1 Date: 2April2012

Reviewer: CPRen

Reviewer's report:

Referee Statement

RE:Zhang et al.,Transferred Tumor suppressor BLU inhibits..

It has been documented that BLU/ZMYND10 is silenced in avariety of cancers

including NPC due to genetic or epigenetic mechanisms .Restored expression of BLU

can inhibit cell growth and proliferation of cancer cells, bu the underlying mechanism

are still unclarified. In this MS, the authors demonstrated that BLU tumor suppressor

gene inhibited NPC cell growth by cell cycle arrest through suppressing JNK activity

and regulating cyclin D1 promoter. Experimental protocols were well controlled, data

presented supports the authors’ observation and the results are reasonable. But several

points listed below should be considered by the authors.

Major Compulsory Revisions

1. Why the authors choose JNK and cyclinD1 as main targets should be addressed

clearly.

As a putative tumor suppressor gene, BLU might code for product to exert

growth inhibition. We have shown in the present MS that the ectopic

expression of BLU inhibits clonogenic growth of cells of NPC and esophageal

cancer origin. Such inhibition and hence tumor suppression could be resulted

from the apoptosis triggering or blocking of cell cycle entry. As stated in the

paper, Ji et al. (ref. Ji et al.， Cancer Res 2002, 62:2715-2720.) evaluated the

apoptotic induction potential of several candidate TSGs, and found that BLU

does not trigger apoptosis in lung cancer lines. In fact, according to our own

observation, the forced expression of BLU fails to induce apoptosis, and there

was not such evidence like blebbing cells or fragemented nuclei occurred when

the cells expressed BLU. We thherefore tested whether BLU regulates cell cycle.

Our data clear showed that it expression arrested cells at G1 phase. We then

looked at the cyclin which regulates G1 phase entry, that is cyclin D1, and its

upstream regulator, JNK. The ref. 11 clearly showed that the promoter of

cyclin D1 gene contains an AP1 binding site, and it is activated by c-fos and

c-jun.

2. Why the authors don’t establish stable BLU-expressed NPC cell line and analyze

the mechanisms? How BLU regulates the activity of JNK and promoter of

cyclinD1 is not well discussed. According to the description in Introduction Part,

BLU seems to be a transcription facto ror a co-transcription factor. However,

according to the results, BLU leads to phosphorylation of c-Jun. Therefore, what

Generated by Foxit PDF Creator © Foxit Software

http://www.foxitsoftware.com For evaluation only.is the direct target of BLU protein and how BLU acts should be elucidated or

discussed. It’s better to use some specific JNK inhibitors to investigate the

mechanisms.

We did not create stable trnasfectant CNE-2 clones for at least two reasons; The

first, The efforts were made previously. Clones were obtained but when

unfrozen the cells survived poorly in comparison with the mock. Similarly, a

laborotory which cloned a TSG, DLC-2 has found that transfection with one of

the DLC-2 isoforms was impossible. The classical trnasfection causes serious

problem in eliminating clones not tolerant to the introduced genes during

antibiotic selection. and, such manipulation is even more unsutiable for a TSG.

The best known system for TSG expression is temperature sensitive (ts) p53.

We have planned to create tet regulatued BLU system using our previously

reported constructs. Secondly, our experiments were conducted to

mechanistically elucidate the result of clonogenic growth inhbition. The cells

were transfected and cultured with G418 for only 2 weeks. Expression of the

target gene for 48 h is sufficient for its biological effects. In relevance to clinical

biotherapy, the target genes delivered by adenovirus or other vectors also

maintain in weeks, not permenantly.

Clearly, the molecule targeted by BLU mediated trnascription repression

remains to be identified. Related findings published todate are that, a

transcription regulatory zinc finger protein modulates JNK-AP1 axis (e. g. Li et

al., Mol Cell Biochem 2008, 310:141-51, cited in the revised version of the MS),

and RASSF1A inhibits JNK-cyclin D1 axis to arrest cell cycle through its

interactions with Ras protein. In view of the stress-repsonsive activation of

BLU and JNK, BLU might transcriptionally regulate an upstream factor on JNK

pathway, and directly modify catalytic activities of JNK.

3. Why the authors use CNE2 cell line? In the second paragraph of Results Part,

what does Results obtained from the experiment using CNE-1 line were Somehow

different” mean?

It was clearly stated in the Methods, and Result and Discussion parts, that the

choice of the cell lines was based on the previous publication from Tao’s group in

Oncogene 2004. The two NPC lines were completely absent of BLU expression.

The optimal dose for BLU Ad infection effciency were tested in the two lines, and

the results were different; the data for CNE-1 was included in supplementary

section.

Minor Essential Revisions

4.In the paragraph of “Cell and plasmid” in Materials and Methods Part,t

Description of the cyclinD1 promoter lacked some details:which portion of

Element was incorporated into the reporte rplasmid?

Generated by Foxit PDF Creator © Foxit Software

http://www.foxitsoftware.com For evaluation only.Thanks for pointing out the problem. In fact the ref. 11. Albanese et al., gave a

clear description of the construct, and we will add it the MS text. The construct

was termed -1745 CD1 LUC, which contains full length cyclin D1 gene promoter,

including two AP1 sites and one Sp-1 element. Overall, the gene spans about 15

kb and has 5 exons; its promoter region has Sp-1 binding sites and no obvious

TATA box,

5.In figure 2, A, B, C, D and E are not indicated.

We are sorry for such problem which was generated due to the software or

internet transfer. As the labels were clear in our draft. The corrections were

made accordingly: Fig 2 A: Result of clonogenic growth inhibtion; Fig. 2B: the

summary of the data in (A). Fig. 2C, the cell cycle profile of mock transfectant of

CNE-2 with vector pCD316; and Fig. 2D, that of BLU transfectant CNE-2.

6.In figure 3D, lane 1, 2, 3, 4 are not indicated.

We have checked the original PowerPoint file, the numbers counted from right

to left were 1, 2, 3, 4, which correspond to the doses of 0, 10, 50, and 100 PFU Ad

BLU.

7.Figure 4 is not described in Legends and Results.

The MS contains only three Figure panel, but contains a supplementary figure.

8.There is[reference???] in Results Part.

We apologize for such problem, and corrected it by adding “[11]”.

9.English writing needs to be improved. Generally speaking, it is better that the

sentences in Results Part are written in past tense.The title is a little confusing.

We have found some mistakes in sentense structure and so on. They have been

corrected after going through the entire paper. Meanwhile, a company Edanz as

suggested by the eitor provided editorial assistance. The tilte has been modified

after the correction and it looks much clear now.

Level of interest: An article whose findings are important to those with closely

related research interests

Quality of written English: Needs some language corrections before being published

Statistical review: Yes, and I have assessed the statistics in my report.

Declaration of competing interests:

I declare that I have no competing interests.

Generated by Foxit PDF Creator © Foxit Software

http://www.foxitsoftware.com For evaluation only.
